# A Novel Bioabsorbable Covered Stent for Advancing Bile Duct Injury Management: A Preclinical Study in a Porcine Model (With Video)

**DOI:** 10.1002/deo2.70162

**Published:** 2025-06-10

**Authors:** Mitsuo Miyazawa, Masayasu Aikawa, Junpei Takashima, Hirotoshi Kobayashi, Takuya Minagawa, Osamu Itano, Shunsuke Ohnishi

**Affiliations:** ^1^ Center for Preventive Medicine International University of Health and Welfare Narita Hospital Narita Japan; ^2^ Department of Gastroenterological Surgery Saitama Medical University International Medical Center Saitama Japan; ^3^ Department of Surgery Teikyo University School of Medicine Mizonokuchi Hospital Kawasaki Japan; ^4^ Department of Surgery International University of Health and Welfare Narita Japan; ^5^ Department of Gastroenterology and Hepatology Hokkaido University Graduate School of Medicine Sapporo Japan

**Keywords:** bile duct injuries, biodegradable materials, cholecystectomy, endoscopy, stents

## Abstract

Self‐expandable metallic and plastic stents have been used for biliary tract injuries, but they are not entirely adequate as treatments. This study investigated the potential of our novel self‐expandable bioabsorbable covered stent (SEBCS) to treat bile duct injuries. We developed a novel SEBCS by covering a self‐expandable bioabsorbable stent with a bioabsorbable tube. Five pigs underwent laparotomy after being placed under general anesthesia. A 5‐mm incision was made in the extrahepatic bile duct, followed by the insertion of an SEBCS. Postoperatively, hepatobiliary enzyme levels were measured. At 10 weeks postoperatively, a histological evaluation of the injured area and cholangiogram were performed. The SEBCS was successfully inserted into the extrahepatic bile ducts of all animals. The histological evaluation at 10 weeks postoperatively showed epithelial regeneration with numerous peribiliary glands, including at the injury site. Cholangiography revealed no stenosis in the injured area. Hematological and biochemical analyses revealed mild elevation of biliary enzyme levels on day 10 postoperatively compared with preoperative levels; these levels returned to preoperative values by week 10. This novel SEBCS technique demonstrated the potential to regenerate bile ducts at the site of extrahepatic bile duct injury and may be a promising endoscopic treatment for biliary tract injuries.

## 1 Introduction

Numerous complications associated with bile duct injury have been reported [1]. Although metal and tube stents have been used as treatment options [2, 3], they do not actively promote tissue repair or regeneration without stenosis. Metal stents, in particular, pose challenges because of their difficult removal and the risk of bile duct stenosis associated with epithelial contusion during extraction [4]. Tube stents require multiple insertion procedures and frequently lead to stenosis at the injury site after removal; therefore, they are suboptimal endoscopic treatment options [5]. The ideal treatment for bile duct injuries that occur during laparoscopic cholecystectomy, whereby bile leaks from the extrahepatic bile duct, involves endoscopic sealing of the bile leak intraoperatively or early postoperatively using a bandage‐like sheet, followed by further repair. Such an approach would facilitate bile duct regeneration and help prevent long‐term narrowing.

Therefore, we developed a bioabsorbable sheet to promote bile duct regeneration [6, 7, 8]. During this study, we investigated whether a self‐expandable bioabsorbable covered stent (SEBCS) comprising a self‐expandable bioabsorbable stent and bioabsorbable sheet could be used as an effective treatment for benign biliary tract injuries.

## 2 Procedure

### 2.1 Bioabsorbable Tube

**FIGURE 1:**
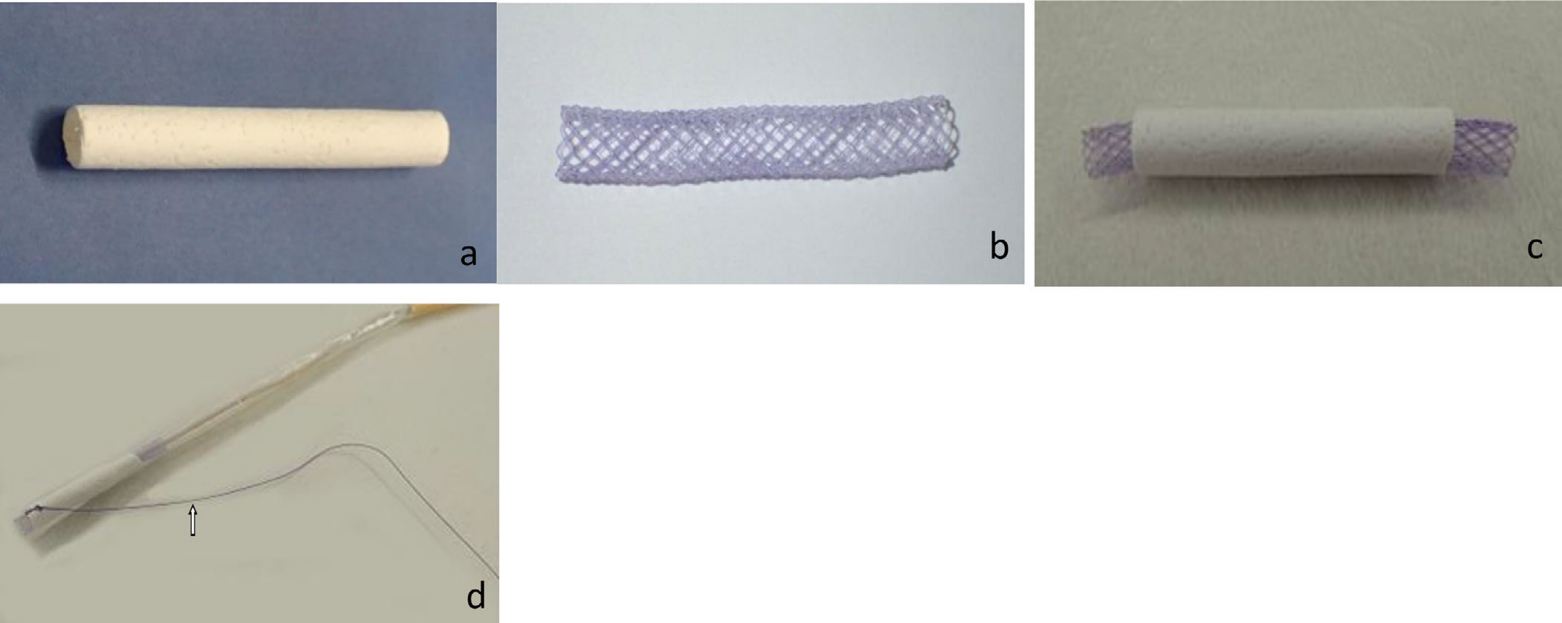
(a) Bioabsorbable tube. This bioabsorbable tube is made of a 50:50 copolymer of lactic acid and caprolactone reinforced with polyglycolic acid fibers designed to allow in vivo absorption within 6 to 8 weeks. In its natural state, it retains its annular shape. (b) Self‐expanding bioabsorbable stent. This stent is made of a 75:25 copolymer of lactic acid and caprolactone and has a complete in vivo degradation period of approximately 20 weeks. It becomes fragile in the presence of digestive fluids. (c) Self‐expanding bioabsorbable covered stents. The self‐expanding bioabsorbable covered stent was created by inserting a self‐expanding bioabsorbable stent into the bioabsorbable tube. (d) Stent insertion introducer. A self‐expanding bioabsorbable covered stent was attached to this introducer and inserted through the papilla of Vater. The stent is secured with a thread that allows its removal (arrow).

The bioabsorbable tube, which forms part of the sheet that covers the SEBCS, is composed of a 50:50 copolymer of lactic acid and caprolactone reinforced with polyglycolic acid fibers (Gunze Co., Kyoto, Japan). Its design allows it to be fully absorbed in vivo within 6–8 weeks. In its natural state, the tube maintains its annular shape (Figure 1a) and has a cylindrical structure with a diameter of 5 mm, length of 5 cm, and wall thickness of approximately 1 mm. With porosity exceeding 95%, the tube is designed to facilitate cell adhesion. It is spongy and easy to handle, does not feel rigid, and quickly regains its original shape after manual compression. The braided glycolic acid fibers reinforce the structure, allowing for surgical suturing without tearing when a needle passes through. During implantation, bile permeates the polymer, resulting in yellowing. Notably, the tube remains impermeable to water and bile, thus preventing leakage [6, 7].

### 2.2 Bioabsorbable Stent

The SEBCS stent is made of a 75:25 copolymer of lactic acid and caprolactone and has a complete in vivo degradation time of approximately 20 weeks (Gunze Co., Kyoto, Japan). When fully expanded, the stent has a diameter of 5 mm and a length of 5 cm (Figure 1b) [9].

### 2.3 Self‐Expandable Bioabsorbable Covered Stent

At the start of the experiment, a bioabsorbable stent was inserted into the bioabsorbable tube, thus forming an SEBCS (Figure 1c).

### 2.4 Experimental Procedures

All experimental procedures involving animals were conducted in accordance with the National Institutes of Health (NIH) guidelines for the care and use of laboratory animals and approved by the Animal Experiment Committee of Saitama Medical University.

Hybrid pigs (*n* = 5) with a weight of 15–25 kg were fasted for 12 hours before surgery. Anesthesia was induced with an intramuscular injection of ketamine (20 mg/kg), followed by an intravenous injection of propofol (1 mg/kg). Then, animals were intubated intratracheally and mechanically ventilated. During surgery, anesthesia was maintained with a continuous infusion of propofol (4 mg/kg/h). A midline incision was made to open the abdomen. The extrahepatic bile duct within the hepatoduodenal mesentery was identified and dissected. A 5‐mm diameter opening was created in the anterior wall of the extrahepatic bile duct at approximately 3 cm from the superior border of the duodenum. Then, a 5‐cm incision was made in the anterior wall of the descending duodenum to expose the papillary region of Vater. The Vater papilla was dilated using Pean forceps. The SEBCS was inserted in the introducer, advanced through the Vater papilla, and positioned so that the opening in the bile duct was centered within it. A balloon catheter was introduced through the Vater papilla and expanded to ensure proper adhesion of the stent to the perforated area. To maintain patency, a 6‐Fr plastic stent was inserted through the Vater papilla as a lost tube to bridge the opening. The proximal end of the lost tube extended into the lumen on the liver side, and the distal end extended 5 cm into the duodenum. A nylon thread attached to the SEBCS (Figure 1d) was passed through the pyloric ring and exited via the anterior wall of the gastric body toward the abdominal wall. Then, the duodenum and abdominal incision were closed with the nylon thread, which was secured to the right side of the abdominal wound before final closure. Postoperatively, the animals were provided with a normal diet and administered antibiotic therapy until postoperative day 3 (Video S1).

On postoperative day 10, using the same anesthesia protocol as that for the initial surgery, the nylon threads that secured the bioabsorbable covered stent were removed, thus allowing the stent to fall into the duodenum from the Vater papilla. Blood samples were collected from the subclavian sinuses.

At postoperative week 10, the pig with the bioabsorbable covered stent was anesthetized according to the initial surgical protocol. The abdomen was opened and gallbladder cholangiography was performed. Blood samples were drawn from the subclavian sinus. A sample of the extrahepatic bile duct was obtained and histological changes at the perforated site were examined.

### 2.5 Measurement of Hepatobiliary Enzymes

Blood samples were collected before stent insertion, 10 days after insertion, and 10 weeks after stent insertion to evaluate the liver function after SEBCS insertion.

### 2.6 Cholangiography (Postoperative Week 10)

Cholangiography was performed to examine bile flow in the bile ducts. A 76% urographin solution was diluted 1:1 with saline and injected into the body of the gallbladder. A C‐arm (OEC One; GE Healthcare Japan) was used to position and capture the entire bile duct.

## 3 Results

### 3.1 SEBCS Insertion

In all pigs in this study (*n* = 5), the SEBCS successfully closed the hole in the extrahepatic bile duct, thus preventing bile leakage during abdominal closure. All pigs with the SEBCS gained weight and survived until they were euthanized. The stent portion of the SEBCS was discharged with the stool from all pigs between 5 and 7 weeks after insertion (at 5 weeks for two pigs, at 6 weeks for two pigs, and at 7 weeks for one pig).

### 3.2 Changes in Hepatobiliary Enzymes Over Time After SEBCS Insertion

On postoperative day 10, an increase in biliary enzyme levels compared to the preoperative values was observed. However, by week 10, these levels returned to the preoperative levels (Table 1).

**TABLE 1:** Biochemical assay results.

	Postoperative day 0	Postoperative day 10	Postoperative week 10
**ALT (IU/L)**	23.0 (19.3–26.6)	31.4 (26.5–36.2)	22.0 (19.6–28.0)
**ALP (IU/L)**	179 (165–192)	253 (236–280)	208 (178–220)
**T‐Bil (mg/dL)**	0.8 (0.7–0.8)	1.0 (0.8–1.1)	0.9 (0.7–1.0)

Values are presented as median (range).

Abbreviations: AL, alkaline phosphatase; ALT, alanine aminotransferase; T‐Bil, total bilirubin.

### 3.3 Gross Findings at 10 Weeks After SEBCS Insertion

Mild adhesions were observed in the abdominal cavity. Additionally, mild adhesions to the surrounding tissue were also seen around the extrahepatic bile duct; however, they were easily detached, The extrahepatic bile duct was identifiable.

### 3.4 Histological Findings at 10 Weeks After SEBCS Insertion

**FIGURE 2:**
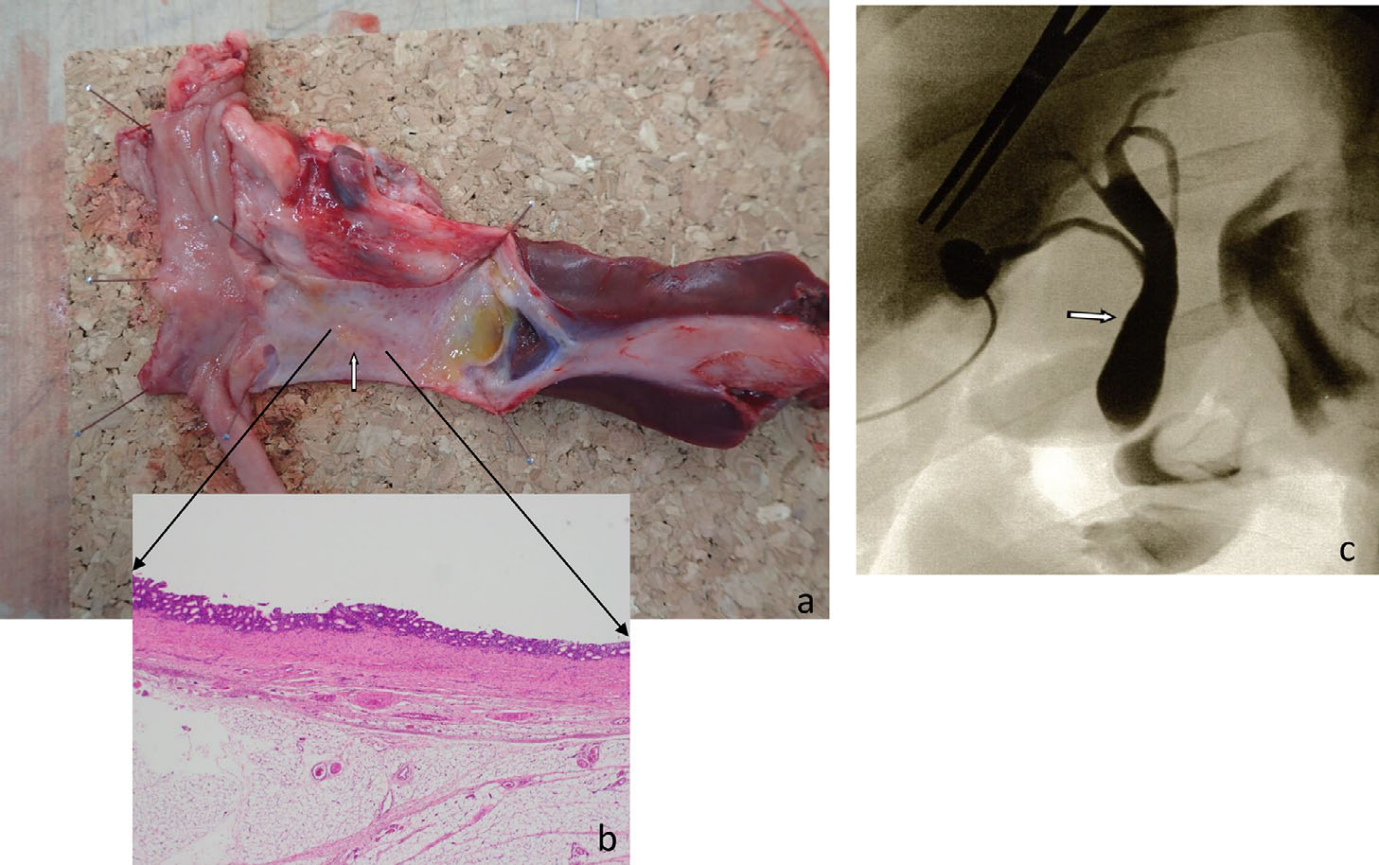
Histological findings 10 weeks after self‐expandable bioabsorbable covered stent (SEBCS) insertion (a and b). (a) Grossly, the perforated area appears slightly reddish compared to the surrounding areas and is identified in the fresh specimen. (b) Histologically, the perforated area is indistinguishable from the surrounding areas. On the bile passage side, the epithelium shows numerous peribiliary glands. Cholangiography 10 weeks after SEBCS insertion (c). The perforated portion of the extrahepatic bile duct is not identified after contrast injection in the gallbladder, and the contrast flows smoothly into the duodenum. Intrahepatic bile duct dilation is not observed.

Gross examination indicated that the ductal structure was maintained in all excised specimens. Signs of bile leakage and peritonitis were not observed in any of the pigs. The perforated area appeared slightly reddish compared to other areas and was identifiable in the raw specimen (Figure 2a). During the histological evaluation, the perforated area was indistinguishable from the surrounding areas. On the biliary transit side, the regenerated epithelium retained numerous peribiliary glands, including those in the perforated area (Figure 2b).

### 3.5 Cholangiography (Postoperative Week 10)

Contrast injected in the gallbladder did not allow identification of the perforated portion of the extrahepatic bile duct. Additionally, the contrast drained smoothly into the duodenum. Intrahepatic bile duct dilation was not observed (Figure 2c).

## 4 Discussion

Intraoperative bile duct injury is common during laparoscopic cholecystectomy and often leads to bile leakage. The findings of this study suggest that the SEBCS can effectively repair injured areas and promote bile duct regeneration. Furthermore, the SEBCS does not cause significant epithelial surface contusions, which are common with self‐expanding metal stents [5]. Additionally, tube stents alone are often insufficient for direct repair of the injured area. The SEBCS has the potential to overcome the limitations of current stents used for bile duct injuries.

Only tubular [10] and reticular [11, 12] bioabsorbable stents without cover sheets have been reported; however, our bioabsorbable stent is covered. Additionally, the use of biodegradable stents to treat bile leakage caused by bile duct injury has been attempted [11, 12]; however, those studies did not use a stent covered with a bioabsorbable sheet such as ours. These results indicate that SEBCS may be a novel treatment for bile duct diseases that is capable of directly repairing bile duct injuries and defects while promoting bile duct regeneration. Specifically, in addition to treating bile leakage after laparoscopic cholecystectomy, the SEBCS may be used to manage bile leakage after refractory hepatobiliary surgery [13] and address the high incidence of bile duct complications associated with liver transplantation [14].

The SEBCS consists of a bioabsorbable stent11 and a bioabsorbable tube. It has a weaker radial force compared to that of metallic stents [15]; therefore, it is expanded by a balloon when inserted in the injured area. This reduced radial force minimizes the risk of epithelial damage during stent removal, thus promoting normal bile duct epithelial regeneration with numerous peribiliary glands at the site and preventing subepithelial connective tissue growth 10 weeks after insertion [16] (Figure 2b). Although the stent is bioabsorbable, complete absorption may require up to 20 weeks. This stent was excreted from all pigs within 5–7 weeks without complications.

Our previous studies of bioabsorbable tubes that cover the SEBCS have shown that bile can permeate the tube without leaking, thereby preventing bile leakage from the injured area [6, 7]. Additionally, the cell adhesion properties are superior to those of other bioabsorbable materials, thus inducing good bile duct regeneration. Additionally, our previous studies demonstrated that bile duct tissue regenerates on the exterior of the tube, suggesting that thicker bile ducts that are less prone to stenosis after treatment will regenerate from the stent [8, 17].

During the experimental procedure, a nylon thread was attached to the SEBCS to assist with removal. At 10 days postoperatively, the nylon thread was pulled toward the duodenum, thus allowing removal of the SEBCS from the bile duct and its placement in the duodenum. This approach was adopted because cholangitis is a complication of bioabsorbable stent treatment [18] and because it has been hypothesized that bile adheres to the stent and induces infection. Additionally, previous studies suggested that fibrous connective tissue forms on the exterior of the stent as a foreign body reaction within 10 days of SEBCS insertion [8, 17], thereby preventing bile leakage from the damaged area. During this study, no hyperplasia of the bile duct epithelium was observed and no complications, such as peritonitis, were noted after SEBCS removal, thus proving the effectiveness of this technique. During actual endoscopic treatment, a nylon thread should be attached to the SEBCS, the thread tip should be maintained in the duodenum, and the SEBCS should be endoscopically withdrawn approximately 10 days after insertion; these procedures are similar to those used to insert an internal stent in the bile duct [19].

With the SEBCS, the outer circumference adhered closely to the hole drilled in the bile duct, thus preventing bile leakage. Although it is not clear whether this procedure ensured complete repair of the damaged area, bile leakage did not occur and the damaged area regenerated. In clinical practice, when inserting the SEBCS endoscopically, a balloon should be inserted in the SEBCS to ensure that the tube portion adheres to the injured area, thus enhancing safety [20].

During this study, the SEBCS was inserted in an open abdomen to simulate the endoscopic insertion technique. After the duodenum was incised, the SEBCS was securely inserted in the papilla of Vater. Following SEBCS insertion, a tube stent was placed in the lumen of the SEBCS as a lost stent to prevent early cholangitis, resulting in mild elevation of biliary enzyme levels 10 days after insertion. In clinical practice, when the SEBCS is inserted endoscopically, a lost stent should be placed in the SEBCS lumen to enhance safety.

The SEBCS in this study was constructed using a bioabsorbable stent that absorbs within approximately 20 weeks and is covered by a tube that fully absorbs within 6–8 weeks; it was removed 10 days after insertion. Before clinical application is possible, further investigations should determine whether the absorption period and treatment techniques of these bioabsorbable materials are optimal for regenerating injured areas without causing stenosis. Additionally, the introducer and endoscopic insertion technique should be considered because the SEBCS is softer than metal and tubular stents.

This study assumed endoscopic insertion of the SEBCS during the early stage of bile duct injury. However, in actual clinical practice, many cases for which insertion is not possible during the early stage of bile duct injury will likely be encountered. This study was limited by its clinical nature and a small sample of animals. Therefore, further investigations of the technique and stent self‐expansion strength are necessary before the SEBCS can be used in clinical practice.

## 5 Conclusion

This study demonstrated that our novel SEBCS facilitated the repair of the injured bile duct without stenosis and promoted bile duct regeneration at the injury site. Although various modifications are necessary before they can be clinically applied as endoscopic treatment of bile duct injuries, we believe that our novel SEBCS can be utilized as part of a novel endoscopic treatment approach for bile duct injuries.

## Conflicts of Interest

The authors declare no conflicts of interest.

## Ethics Statement

Approval of the research protocol by the institutional review board: Institutional review approval was obtained from the Ethics Committee of Saitama Medical University.

## Consent

N/A.

## Clinical Trial Registration

SMU590.

## Supporting information




**VIDEO S1** Insertion of a self‐expanding bioabsorbable covered stent
